# The Contribution of Emotional Intelligence to Career Success: Beyond Personality Traits

**DOI:** 10.3390/ijerph16234809

**Published:** 2019-11-29

**Authors:** Itziar Urquijo, Natalio Extremera, Garazi Azanza

**Affiliations:** 1 Faculty of Psychology and Education, University of Deusto, 48007 Bilbao, Spain; itziar.urkijo@deusto.es; 2 Faculty of Psychology, University of Málaga, 29016 Málaga, Spain; nextremera@uma.es; 3 Faculty of Social and Human Sciences, University of Deusto, 48007 Bilbao, Spain

**Keywords:** emotional intelligence, career success, big five personality traits, proactive personality, early and later career, sociodemographic factors

## Abstract

This study sought to investigate the role of emotional intelligence in both extrinsic and intrinsic career success in early and later career stages. Specifically, we examined the predictive and incremental validity of emotional intelligence in career success after controlling for personality factors in a sample of 271 graduates. When analyzing extrinsic career success, regression analyses revealed that demographic variables, such as gender, age, area of study and career stage, and the variable of proactive personality, were related to salary. When the dependent variable was job satisfaction, emotional intelligence acted as a strong predictor, even when personality traits and proactive personality were controlled. These findings provide preliminary evidence that emotional intelligence is a relevant addition to guide the achievement of career success. Finally, limitations of the results and implications of these findings are discussed.

## 1. Introduction

Achieving work–life balance and general life satisfaction has become one of the most relevant challenges for workers nowadays [1]. Objective criteria of career success, such as salary, result in higher socioeconomic status and allow people to fulfil their personal responsibilities [2], thus enabling work–life balance and general life satisfaction. However, in order to examine career success, the subjective factors that make employees feel satisfied should also be considered, particularly in the current digital era, which has drastically changed the features of work and the way in which people communicate to each other in organizations, reducing and sometimes hindering social interactions [3]. The need to examine both objective and subjective factors of career success is even more relevant in the Spanish context, where both job and financial satisfaction are lower than the European Union average [4].

In this context, identifying predictors of career success and establishing the concept itself is gaining importance in vocational and organizational career research [5,6]. In the constant search for comprehensive models of career success, researchers have concluded that it is necessary to consider the concept from both an extrinsic and intrinsic perspective [7]. Extrinsic career success has been understood as an objective and externally visible criteria [8] and intrinsic success as subjective feelings of accomplishment and satisfaction with one’s career [9]. Although recent studies have provided important insights into the causes of career success, there is room for further development in the area of individual differences [10,11]. Moreover, there is a substantial body of research pointing to affective and personal components as being key to job outcomes [12,13,14].

Traditionally, the widely recognized Big Five personality traits have been used to explain career success [15,16]. This refers to unchanging traits such as extraversion, neuroticism, openness, conscientiousness and agreeableness [17]. To a certain extent, personality influences the way in which individuals behave in the workplace and their performance at work [18]. For example, those individuals who show higher levels of conscientiousness have more positive feelings about their job and extroverted workers are more involved in workplace social networks [16]. Thus, as personality determines employees’ behavior, some authors have identified it as a factor contributing unique variance to explaining career success [19].

In recent years, the factor of proactive personality, defined as one’s dispositional tendency to take initiative in a variety of situations, has gained importance [20]. Relatedly, individuals’ proactivity or capacity to intentionally change different circumstances is closely related to the Big Five personality traits [21]. Further, having a proactive personality has been positively associated with both objective and subjective career success, that is, proactive people are better able to identify positive opportunities and persevere until they achieve them [22,23].

Given that personality traits are static dispositional characteristics, various studies have gone further and have added personal resources variables while controlling them [24]. Among these variables, evidence is accumulating in relation to the association of emotional intelligence with relevant aspects of the occupational environment [13,25,26,27]. In particular, those individuals with higher emotional intelligence tend to perceive more job success [28], experiment more at work [29] and engage in fewer counterproductive work behaviors [30]. In addition, researchers have argued that individuals with high levels of emotional intelligence have stronger engagement in their work than their colleagues [31,32]. In summary, emotionally intelligent employees are able to reduce negative job outcomes and experience a positive emotional state because of their job evaluations [33,34].

Emotional intelligence has received much attention in the research literature, including discussion of the difference between ability-based and trait-based emotional intelligence [35]. On the one hand, ability-based emotional intelligence is conceptualized as the capacity to perceive and assimilate emotions, understand the implications of those emotions and manage them [36]. On the other hand, trait-based emotional intelligence is considered to be a constellation of emotional self-perceptions located at the lower levels of personality [37]. It has been concluded that both models can be useful in the prediction of relevant workplace success outcomes [38]. According to some authors [26], Emotional intelligence (EI) ability might be evaluated using both performance and self-report ability measures. In fact, there also exist self-report EI ability measures that have been designed for evaluating the core emotional abilities proposed by Mayer and Salovey’s [36] conceptualization of EI, being the Wong and Law Emotional Intelligence Scale (WLEIS) [39]—one of the most commonly used instrument in the workplace setting [40]. Further, self-report EI instruments such as WLEIS are practical because they are relatively short and easy to administer, which might be more useful for survey purposes when time is limited [41]. Therefore, in line with prior research examining predictive and incremental validity in managerial studies [41], a self-report ability EI test was used.

There is a consensus that emotional intelligence and personality overlap on some levels [42,43]. Thus, considering all the above mentioned factors, it is not surprising that researchers have started to consider both factors when explaining career success [44]. Including personality factors as controlled variables is therefore a stringent test supporting the relevance of emotional intelligence, independent from other theoretically and empirically robust predictors. It is also relevant to confirm whether there is any modifiable variable beyond those stable characteristics that can be developed. To some extent, emotional intelligence and personality may operate similarly in terms of their significant relationships with career success indicators.

Previous research has reinforced and extended the idea of the important impact of ability and personality predictors on early career success. However, some aspects are still unclear. For example, Rode et al. [28] examined the effects of the mentioned variables, controlling for gender and being in an early career stage, but without controlling for age, area of study or later career stage. This acquired real prominence to the extent that these factors were critical to determining career success. On the one hand, studies like the aforementioned [28] have suggested that personality has a stronger effect than ability in the first two years of a worker’s career and have emphasized the need for further research to consider the dimension of later career stage. On the other hand, age has been found to have strong implications for career success in several ways because of its relationship with maturity and experience [45,46]. Finally, area of study should also be considered because, as the official Spanish Statistics Institute [47] has indicated, there is a significant economic gap between different sectors.

Based on these limitations, the present study expands the existing research in several ways. Firstly, the study-wide sample considered both early and later career success. Secondly, graduates from different areas of study participated, including students from the fields of Science, Social and Legal Sciences, Health Sciences, Arts and Humanities and Engineering and Architecture. Thirdly, age was controlled to increase the descriptive value of the analysis.

This study used hierarchical regression analysis to assess the predictive role of emotional intelligence, proactive personality and personality factors on career success in both early and later career stages, while controlling for age, gender and area of study. Based on previous studies, we proposed the following hypotheses: that, after controlling for the effects of Big Five personality factors, age, gender, area of study, and proactive personality, (1) emotional intelligence would be positively associated with career success (given extrinsic and intrinsic factors); and (2) emotional intelligence would significantly predict career success (given extrinsic and intrinsic factors).

## 2. Materials and Methods

### 2.1. Participants

Two hundred and seventy-one working graduates (179 women, 92 men) completed our test voluntarily. These participants were recruited using an online platform for graduates from the University of Deusto. On this platform, former students from different areas of study can access job offers and participate in continuous learning processes. Initially, a sample of four hundred graduates took part in the general research project, but for this study, data was collected only from those participants who were working when the questionnaire was administered. Regarding career stage, individuals in the first two years of their careers (37.9 %) were considered ‘early career’, and individuals with more than two years of work experience (62.1%) were considered ‘later career’, consistent with the ranges used in previous studies [48]. Participants’ average age was 31.48 years (SD = 8.47 years, range = 22–60 years). Graduates came from the fields of Sciences (19.9%), Social and Legal Sciences (12.9%), Health Sciences (11.4%), Arts and Humanities (21%) and Engineering and Architecture (34.7%). A sample characteristics summary by career stage can be found in Table 1.

### 2.2. Measures

**Emotional intelligence.** The Spanish version [39] of the Wong and Law Emotional Intelligence Scale (WLEIS) [49] was used to assess self-perceived emotional intelligence. The scale consists of 16 brief statements across four dimensions: self-emotion appraisal, appraisal of others’ emotions, use of emotion and regulation of emotion. The scale includes items such as “I always know whether or not I am happy” or “I can always calm down quickly when I am very angry”. Each item is scored on a 5-point scale (1 = strongly disagree, 3 = neutral, and 5 = strongly agree). The WLEIS elicits a global emotional intelligence score, with a higher score indicating greater emotional intelligence. The Spanish version of the WLEIS has been proven to have good validity and reliability in Spanish populations [50,51]. In this study, the scale had satisfactory alpha coefficients (see Table 2).

**Personality traits.** Personality was assessed using the Spanish version of the short form of Goldberg’s bipolar adjectives [52,53]. This questionnaire includes 25 items and participants indicate the extent of their agreement with pairs of adjectives rated on a 9-point Likert scale. The factors measured are Neuroticism, Extraversion, Openness, Agreeableness and Conscientiousness, consistent with the Big Five dimensions [54]. This version has been shown to have good validity and reliability in Spanish populations [55].

**Proactive personality.** A shortened version of Bateman and Crant’s original scale was used to measure the proactivity of participants’ personalities [20,22]. This scale has 10 items scored on a 7-point agreement scale ranging from 1 (strongly disagree) to 7 (strongly agree). It includes statements such as, “If I believe in an idea, no obstacle will prevent me from making it happen”. The English version of the scale [22] was translated into Spanish and back-translated into English through collaboration with local English and Spanish people.

**Career success criteria.** Following Judge et al.’s [56] approach, both extrinsic and intrinsic measures of career success were used. Extrinsic success was measured based on salary, restricted to the range presented by De Haro & Castejón [44]. Participants’ monthly salary was divided into seven categories: (1) Less than 600€, (2) between 600 and 1000€, (3) between 1000 and 1200€; (4) between 1200 and 1500; (5) between 1500 and 1800€; (6) between 1800 and 2000€, and (7) more than 2000€. Intrinsic success, based on overall job satisfaction, was measured using five items taken from the Brayfield and Rothes job satisfaction scale [57]. The included items were “I feel fairly well satisfied with my present job”, “Most days I am enthusiastic about my work”, “Each day of work seems like it will never end” (reverse scored), “I find real enjoyment in my work”, and “I consider my job rather unpleasant” (reverse scored). This scale was scored on a 10-point Likert scale ranging from 0 (strongly disagree) to 10 (*strongly agree*). This scale showed satisfactory internal consistency, convergent validity and an acceptable divergent validity [58].

**Control variables**. We controlled for variables, such as age, gender, area of study (Sciences, Arts and Humanities, Engineering and Architecture, Social and Legal Sciences, and Health Sciences) and career stage, differentiating between early and later career based on the length of participants’ post-university careers.

### 2.3. Procedure

Study participants were university graduates who were recruited through the aforementioned University of Deusto alumni platform. Participants completed a multi-section online survey that took approximately 20 min. Participants were informed of the confidentiality of their data and all ethical requirements were respected. In exchange for their participation in the study, each subject had the option to participate in a variety of non-economic raffles.

## 3. Results

Descriptive statistics and intercorrelations between the variables are depicted in Table 2. On the one hand, analysis of the variable of emotional intelligence showed a positive significant correlation with age (*r* = 0.19, *p* < 0.01), extraversion (*r* = 0.35, *p* < 0.01), agreeableness (*r* = 0.35, *p* < 0.01), conscientiousness (*r* = 0.30, *p* < 0.01), openness (*r* = 0.30, *p* < 0.01), proactive personality (*r* = 0.48, *p* < 0.01), salary (*r* = 0.16, *p* < 0.01) and job satisfaction (*r* = 0.29, *p* < 0.01). On the other hand, emotional intelligence had a negative significant correlation with neuroticism (*r* = − 0.40, *p* < 0.01). Regarding the relationship between personality traits and career success, we found that only neuroticism was negatively related with salary (*r* = − 0.12, *p* < 0.05). Extraversion and conscientiousness were positively correlated with job satisfaction, while neuroticism was negatively correlated to it. Finally, as Table 2 shows, proactive personality was positively correlated to both career success aspects, salary (*r* = 0.15, *p* < 0.01) and job satisfaction (*r* = 0.22, *p* < 0.01).

As we see in Table 3, two hierarchical multiple regressions were conducted to examine the predictive and incremental validity of emotional intelligence over personality factors and proactive personality for both salary and job satisfaction. Gender, age, area of study, and career stage were entered in Step 1 to control for their confounding effects, followed by personality traits in Step 2, proactive personality in Step 3, and emotional intelligence entered simultaneously in Step 4.

In the first model, with salary as the dependent variable, age, gender, education, and career stage appeared to be significant factors and, thus, added significant variance (∆R² = 0.45). Personality traits, however, did not provide any significant factor or variance for Step 2 (∆R² = 0.00). Proactive personality, in turn, appeared as a significant factor (β = 12, *p* = 0.03) and in Step 3 added significant variance (∆R² = 0.009). Finally, emotional intelligence did not account for any significant variance (β = 0.08, *p* = 0.14). Thus, the proposed first step explained a wide range of the dependent variables (total adjusted *R*² = 0.44), but mainly those represented by demographic factors and by proactive personality in a minor dimension.

In the second model, with job satisfaction as the dependent variable, we undertook the same steps. No socio-demographic factors explained the variance in Step 1 (∆R² = 0.01). In Step 2, a significant variance was explained (∆R² = 0.06) by neuroticism (β = −0.22, *p* = 0.00). When proactive personality was included in the next step, neuroticism (β = −0.21, *p* = 0.00) and proactive personality (β = 0.20, *p* = 0.00) were the only significant factors—proactive personality, positively and neuroticism, negatively—and a significant variance appeared (∆R² = 0.03). Finally, emotional intelligence added a significant variance (∆R² = 0.02) and acted as a significant and explicative factor, even when the control variables, personality traits and proactive personality, were included.

## 4. Discussion 

The purpose of the present study was to investigate the predictive and incremental validity of emotional intelligence over personality factors and proactive personality variables for career success (salary and job satisfaction) in a sample of early and later career graduates. Our findings confirmed the previously reported relationships between emotional intelligence and career success in different populations [44,59,60]. Our results also went beyond these previous findings, including some aspects that were not controlled in previous studies.

Correlational analyses showed that emotional intelligence was positively corelated to both salary and job satisfaction. Regarding salary, our results were similar to the results of De Haro and Castejón [44], who found a positive correlation with emotional repair. The results generated by the relationship between emotional intelligence and job satisfaction were consistent with those found by previous studies [25,60,61]. In addition, the results indicated a significant negative relationship between neuroticism and both salary and job satisfaction, in line with other studies [62,63,64]. However, a positive relationship between extraversion, conscientiousness, agreeableness, and job satisfaction was found, reinforcing the findings of previous research [65]. Consistent with earlier studies, proactive personality was positively related to salary [66] and job satisfaction [67,68].

Regression analyses from our study related to salary indicated that the proposed model sufficiently explained the dependent variable, but not in the expected way. The relevance of emotional intelligence was not evident in the model, in contrast to results reported in other studies, where emotional intelligence appeared as a predictor of salary [44]. However, our findings were in line with other relevant research that found no relationship when controlling for other confounding variables [28]. In De Haro and Castejón’s [44] study, neither gender nor age were controlled, and therefore future research should seek to replicate these findings with these classic sociodemographic variables included. In the study of Rode et al. [28], when gender was included in the model, the effect of emotional intelligence disappeared, in line with our findings. However, salary was largely explained by sociodemographic variables, such as gender, age, area of study, and career stage, and to a lesser extent by proactive personality. With regard to gender, this significance may have various explanations, such as participants choosing one area of study instead of another or having the capability to establish a professional network [69,70]. For example, women and men are traditionally considered to make different educational decisions; while men tend to choose more prestigious academic pathways, women tend to select social and human services fields, which are often the most poorly paid [71]. This trend was also observed in our sample: 26% of women graduated in Arts and Humanities, and 16.2% Social and Legal Sciences, whereas only 8.7% and 7.6% of men, respectively, held degrees from those fields. Given these choices, men likely benefit from more opportunities for contact with powerful networks, while women likely do not [72]. Further, the cultures of many organizations render salary negotiations inequitable between women and men, with women being more vulnerable to poor working conditions [73].

Age and career stage results were consistent with the meta-analysis of predictors of career success, which emphasized the importance of age and promotion in the workplace for higher salary [74]. This might be related to the fact that job experience has been linked to promotion opportunities and the acquisition of economic benefits [75,76]. Moreover, having a job tenure profile has been found to lead to easier promotions and, therefore, to wage growth [77]. In addition, having a proactive personality had an explanatory role, which could be explained by the ability to persist in the face of career obstacles and achieve a subsequent salary increase. Thus, individuals with proactive personalities may receive more promotions during their careers, influencing their salary attainment [22].

Another finding from the study showed that emotional intelligence maintained its explanatory power over job satisfaction beyond and above other well-known factors such as proactive personality and neuroticism. That is, people with high levels of emotional intelligence were more likely to have better job satisfaction, enabling a greater sense of accomplishment in their careers. This might be due to the fact that people with high emotional intelligence are more likely to work in teams [78,79], achieving competitive advantages and becoming more motivated [80]. In addition, individuals with high emotional intelligence have the ability to adapt their internal feelings and emotional responses and, in turn, experience more job satisfaction [81].

Our results revealed a negative relationship between neuroticism and job satisfaction, in line with other frameworks where the five-factor model was studied as a dispositional source of job satisfaction [82]. Part of this effect may be due to the fact that neuroticism is a broad construct, defined as the opposite of emotional stability, which, in fact, determines job satisfaction. Extending the study of personality factors, proactive personality appeared as a significant mechanism in the relationship with job satisfaction, supporting the ideas of Li et al. [67]. Similarly, this could be linked with the idea that individuals with proactive personalities desire to take action and promote positive changes in their work environment, which may be related to the feeling of satisfaction in the workplace [83].

The present study was certainly not without limitations. The first limitation was the small sample size. It would be interesting to replicate this research using a bigger sample size to increase the explanatory power of the study. In addition, the sample had a larger number of women than men. Future studies with gender heterogeneity would address this issue. The sample also consisted of only university graduates, so future research should focus on expanding the sample to a generalized population. The second limitation was that our data relied on self-reported measures and may have been susceptible to several biases, such as limited self-knowledge or social desirability. Performance measures such as the Mayer–Salovey–Caruso Emotional Intelligence Test (MSCEIT) [84] could be used to minimize the influence of subjectivity by providing less interpretative results. The use of online methods to collect information may have lowered the response control; it may therefore be preferable to collect data using other approaches. The third limitation was the cross-sectional design, which limited the study because the cause-effect inferences were difficult to draw. New research directions might be possible if longitudinal and experimental studies were used. Finally, in this study, intrinsic career satisfaction was assessed through job satisfaction, which, as stated by Judge and Kammeyer-Mueller [18], is usually “directed around one’s immediate emotional reactions to one’s current job” (p. 60). Therefore, future studies should more specifically measure intrinsic career satisfaction, including both past and future work history.

Despite its limitations, the current study provides a promising addition to frameworks in the field of career success, extending previous relevant research. It represents the first attempt to examine the predictive effect of emotional intelligence in both early and later career success, even when controlling for Big Five personality traits and proactive personality and considering relevant demographic variables such as gender, age and career stage. Our findings suggest that emotional intelligence may predict some intrinsic career success outcomes.

Finally, the present study provides insights that could guide career success. The development of emotional intelligence as a personal resource to achieve career success above personality and proactive personality opens a new line of research, which could examine emotional intelligence programs as a significant tool for career counsellors working with people transitioning into the workforce [85,86]. Future work in this area should not focus on dispositional personality traits but should instead seek to develop an understanding of emotional intelligence. Although our results provide some interesting preliminary evidence concerning the predictive validity of emotional intelligence over some specific aspects of career success, these findings should be interpreted with caution. The effect of emotional intelligence was modest in predicting intrinsic career success. However, small or weak effects should not be dismissed when considering key outcomes that are influenced by different factors [87]. In competitive settings such as workplaces, a small advantage can make a big difference, and assessing and training emotional skills is likely to be most useful in these contexts [88]. Therefore, pending replication, our findings suggest potential avenues for further developing emotional intelligence approaches aimed at improving some aspects of career success in employees.

## 5. Conclusions

The present study analyzed the predictive and incremental validity of emotional intelligence in career success after controlling for personality factors. Our results revealed that demographic variables, such as gender, age, area of study and career stage, and the variable of proactive personality, were related to salary. When the dependent variable was job satisfaction, emotional intelligence acted as a strong predictor, even when personality traits and proactive personality were controlled. These findings provide preliminary evidence that emotional intelligence is a relevant addition to guide the achievement of career success.

## Figures and Tables

**Table 1:** Descriptive statistics for early and later career.

		Early Career	Later Career
**Gender**	Women	72.8%	62%
	Men	27.2	38%
**Age**	M	26.5	34.7
	SD	5.51	9.13
**Educational area**	Sciences	19.3%	21.4%
	Social and Legal Sciences	13.9%	12.6%
	Arts and Humanities	21.7%	19.4%
	Engineering and Architecture	35.5%	32%
	Health Sciences	9.6%	14.6%

**Table 2:** Descriptive statistics and partial correlations and Cronbach’s Alpha internal consistency reliabilities between measures.

	1	2	3	4	5	6	7	8	9	10	11	12
1. Gender	-											
2. Age	0.20**											
3. Career stage	0.11	0.47**	-									
4. Extraversion	−0.13*	0.02	0.03									
5. Agreeableness	0.16**	0.16**	−0.02	0.44**								
6. Conscientiousness	−0.17**	0.05	−0.02	0.49**	0.41**							
7. Neuroticism	−0.21**	−0.11	−0.03	−0.39**	−0.27**	−0.23**	-					
8. Openness	0.13*	0.04	−0.11	0.37**	0.42**	0.32**	−0.31**	-				
9. Proactive personality	0.10	0.15*	0.02	0.26**	0.55*	0.37**	−0.22**	0.30**	-			
10. Emotional intelligence	0.06	0.19**	0.05	0.35**	0.35**	0.30**	−0.40**	0.30**	48**	-		
11. Salary	0.35**	0.56**	0.47**	−0.02	0.06	−0.00	−0.12*	−0.05	0.15**	0.16**	-	
12. Job satisfaction	−0.04	0.02	0.02	0.16**	0.11	0.14*	−0.23**	0.10	0.22**	0.29**	0.13*	-
M		31.48		7.22	7.03	6.25	6.11	7.19	5.02	5.25	5.20	4.5
SD		8.47		1.13	1.11	1.55	1.44	1.20	0.88	0.81	2.08	1.45
α				0.79	0.80	0.90	0.83	0.81	0.88	0.92	-	0.87

Note. N = 271. * *p* < 0.05, ** *p* < 0.01.

**Table 3:** Results of hierarchical multiple regression analyses of career success.

	R²	F	Unstandardized Coefficients		Standardized Coefficients	∆R²
			B	Standard Error	β	
**Salary**						
*Demographic variables*	0.45	53.32				0.44**
Gender			0.80	0.24	0.18**	
Age			0.09	0.01	0.39**	
Area of study			0.29	0.08	0.18**	
Career stage			1.06	0.22	0.25**	
*Personality traits*	0.45	23.40				0.00
Extraversion			−0.04	0.11	−0.02	
Agreeableness			−0.15	0.12	−0.08	
Conscientiousness			0.00	0.08	0.00	
Neuroticism			−0.00	0.08	−0.00	
Openness			−0.00	0.09	−0.00	
*Proactive personality*	0.46	21.79	0.24	0.16	0.09	0.01*
*Emotional intelligence*	0.46	20.10	0.22	0.15	0.08	0.00
**Job satisfaction**						
*Demographic*	0.01	0.89				0.01
Gender			−0.18	0.21	−0.06	
Age			−0.01	0.01	−0.04	
Area of study			−0.08	0.07	−0.07	
Career stage			0.08	0.20	0.02	
*Personality traits*	0.07	2.39				0.06**
Extraversion			0.00	0.09	0.00	
Agreeableness			−0.08	0.10	−0.06	
Conscientiousness			0.02	0.07	0.03	
Neuroticism			−0.17	0.07	−0.17*	
Openness			−0.05	0.08	−0.04	
*Proactive personality*	0.10	2.97	0.26	0.14	0.14	0.03**
*Emotional intelligence*	0.12	3.30	0.32	0.13	0.18*	0.02**

Note. N = 271. * *p* < 0.05, ** *p* < 0.01.

## References

[1] Sirgy M.J., Lee D.-J. (2018). Work-life balance: An integrative review. Appl. Res. Qual. Life.

[2] Parkes L.P., Langford P.H. (2008). Work–life bal ance or work–life alignment? A test of the importance of work-life balance for employee engagement and intention to stay in organisations. J. Manag. Organ..

[3] Abdel-Aziz A.A., Abdel-Salam H., El-Sayad Z. (2016). The role of ICTs in creating the new social public place of the digital era. Alex. Eng. J..

[4] EUROSTAT Quality of Life. https://ec.europa.eu/eurostat/cache/infographs/qol/index_en.html.

[5] Cha J., Kim S.J., Beck J., Knutson B.J. (2017). Predictors of career success among lodging revenue managers: Investigating roles of proactive work behaviors. Int. J. Hosp. Tour. Adm..

[6] Gilar R., de Haro J.M., Castejon J.L. (2015). Individual differences in predicting occupational success: The effect of population heterogeneity. Revista de Psicología del Trabajo y de las Organizaciones.

[7] Spurk D., Hirschi A., Dries N. (2019). Antecedents and outcomes of objective versus subjective career success: competing perspectives and future directions. J. Manag..

[8] Jaskolka G., Beyer J.M., Trice H.M. (1985). Measuring and predicting managerial success. J. Vocat. Behav..

[9] London M., Stumpf S.A. (1982). Managing Careers.

[10] Cabanas E., Sánchez-González J.-C. (2016). Inverting the pyramid of needs: Positive psychology’s new order for labor success. Psicothema.

[11] Ramaswami A., Dreher G.F., Bretz R., Wiethoff C. (2010). Gender, mentoring, and career success: The importance of organizational context. Pers. Psychol..

[12] Bargsted M. (2017). Impact of personal competencies and market value of type of occupation over objective employability and perceived career opportunities of young professionals. Revista de Psicología del Trabajo y de las Organizaciones.

[13] Levitats Z., Vigoda-Gadot E. (2017). Yours emotionally: How emotional intelligence infuses public service motivation and affects the job outcomes of public personnel. Public Adm..

[14] Merino M.D., Privado J., Arnaiz R. (2019). Is There Any Relationship between Unemployment in Young Graduates and Psychological Resources? An Empirical Research from the Conservation of Resources Theory. Revista de Psicología del Trabajo y de las Organizaciones.

[15] Sundstrom E.D., Lounsbury J.W., Gibson L.W., Huang J.L. (2016). Personality Traits and Career Satisfaction in Training and Development Occupations: Toward a Distinctive T&D Personality Profile. Hum. Resour. Dev. Q..

[16] Sutin A.R., Costa P.T., Miech R., Eaton W.W. (2009). Personality and career success: Concurrent and longitudinal relations. Eur. J. Personal..

[17] Rantanen J., Metsäpelto R.L., Feldt T., Pulkkinen L., Kokko K. (2007). Long-term stability in the Big Five personality traits in adulthood. Scand. J. Psychol..

[18] Judge T.A., Kammeyer-Mueller J.D., Peiperl L., Gunz H. (2007). Personality and career success. Handbook of Career Studies.

[19] Judge T.A., Higgins C.A., Thoresen C.J., Barrick M.R. (1999). The Big Five personality traits, general mental ability, and career success across the life span. Pers. Psychol..

[20] Bateman T.S., Crant J.M. (1993). The proactive component of organizational behavior: A measure and correlates. J. Organ. Behav..

[21] Bakker A.B., Tims M., Derks D. (2012). Proactive personality and job performance: The role of job crafting and work engagement. Hum. Relat..

[22] Seibert S.E., Crant J.M., Kraimer M.L. (1999). Proactive personality and career success. J. Appl. Psychol..

[23] Turban D.B., Moake T.R., Wu S.Y.-H., Cheung Y.H. (2017). Linking extroversion and proactive personality to career success: The role of mentoring received and knowledge. J. Career Dev..

[24] Stoll G., Rieger S., Lüdtke O., Nagengast B., Trautwein U., Roberts B.W. (2017). Vocational interests assessed at the end of high school predict life outcomes assessed 10 years later over and above IQ and Big Five personality traits. J. Personal. Soc. Psychol..

[25] Kafetsios K., Zampetakis L.A. (2008). Emotional intelligence and job satisfaction: Testing the mediatory role of positive and negative affect at work. Personal. Individ. Differ..

[26] Miao C., Humphrey R.H., Qian S. (2017). A meta-analysis of emotional intelligence and work attitudes. J. Occup. Organ. Psychol..

[27] Poon J.M.L. (2004). Career commitment and career success: Moderating role of emotion perception. Career Dev. Int..

[28] Rode J.C., Arthaud-Day M.L., Mooney C.H., Near J.P., Baldwin T.T. (2008). Ability and personality predictors of salary, perceived job success, and perceived career success in the initial career stage. Int. J. Sel. Assess..

[29] Kafetsios K., Nezlek J.B., Vassilakou T. (2012). Relationships between leaders’ and subordinates’ emotion regulation and satisfaction and affect at work. J. Soc. Psychol..

[30] Krishnakumar S., Hopkins K., Robinson M.D. (2017). When feeling poorly at work does not mean acting poorly at work: The moderating role of work-related emotional intelligence. Motiv. Emot..

[31] De Clercq D., Bouckenooghe D., Raja U., Matsyborska G. (2014). Servant leadership and work engagement: the contingency effects of leader-follower social capital. Hum. Resour. Dev. Q..

[32] Rodriguez G., López-Pérez B., Ferreo M., Fernandez E., Fernández J. (2017). Impact of the Intensive Program of Emotional Intelligence (IPEI) on middle man-agers’ emotional intelligence. Psicothema.

[33] Meisler G., Vigoda-Gadot E. (2014). Perceived organizational politics, emotional intelligence and work outcomes. Pers. Rev..

[34] Ouyang Z., Sang J., Li P., Peng J. (2015). Organizational justice and job insecurity as mediators of the effect of emotional intelligence on job satisfaction: A study from China. Personal. Individ. Differ..

[35] Petrides K.V., Pita R., Kokkinaki F. (2007). The location of trait emotional intelligence in personality factor space. Br. J. Psychol..

[36] Mayer J.D., Salovey P., Salovey P., Sluyter D. (1997). What is Emotional Intelligence.

[37] Petrides K.V., Furnham A. (2000). On the dimensional structure of emotional intelligence. Personal. Individ. Differ..

[38] Joseph D.L., Newman D.A. (2010). Emotional intelligence: An integrative meta-analysis and cascading model. J. Appl. Psychol..

[39] Extremera N., Rey L., Sánchez-Álvarez N. (2019). Validation of the Spanish version of the Wong Law Emotional Intelligence Scale (WLEIS-S). Psicothema.

[40] Wong C.S. (2015). Emotional Intelligence at Work: 18-Year Journey of a Researcher.

[41] Law K.S., Wong C.-S., Song L.J. (2004). The construct and criterion validity of emotional intelligence and its potential utility for management studies. J. Appl. Psychol..

[42] Amelang M., Steinmayr R. (2006). Is there a validity increment for tests of emotional intelligence in explaining the variance of performance criteria?. Intelligence.

[43] Extremera N., Fernández-Berrocal P. (2005). Perceived emotional intelligence and life satisfaction: Predictive and incremental validity using the Trait Meta-Mood Scale. Personal. Individ. Differ..

[44] De Haro J.M., Castejón J.L. (2014). Does trait emotional intelligence predict unique variance in early career success beyond IQ and personality?. J. Career Assess..

[45] Bal P.M., van Kleef M., Jansen P.G.W. (2015). The impact of career customization on work outcomes: Boundary conditions of manager support and employee age. J. Organ. Behav..

[46] Van der Heijden B.I.J.M., de Lange A.H., Demerouti E., Van der Heijde C.M. (2009). Age effects on the employability–career success relationship. J. Vocat. Behav..

[47] INE (2019). Salario Medio Anual por Sectores de Actividad Económica y Periodo. http://www.ine.es/jaxiT3/Tabla.htm?t=10911.

[48] Cohen A. (1991). Career stage as a moderator of the relationships between organizational commitment and its outcomes: A meta-analysis. J. Occup. Psychol..

[49] Wong C.-S., Law K.S., Seibert S., Kraimer M., Crant M. (2002). The effects of leader and follower emotional intelligence on performance and attitude. Leadersh. Q..

[50] Lopez-Zafra E., Gartzia L. (2014). Perceptions of gender differences in self-report measures of emotional intelligence. Sex Roles.

[51] Pena M., Extremera N. (2012). Inteligencia emocional percibida en el profesorado de Primaria y su relación con los niveles de burnout e ilusión por el trabajo (engagement). Rev. Educ..

[52] García O., Aluja A., Garcia L.F. (2004). Psychometric properties of goldberg’s 50 personality markers for the big five model1 zuckerman-kuhlman-aluja personality questionnaire as a predictor of pathological personality dimensions (PID5). Eur. J. Psychol. Assess..

[53] Goldberg L.R. (1992). The development of markers for the Big-Five factor structure. Psychol. Assess..

[54] Smith D.R., Snell W.E. (1996). Goldberg’s bipolar measure of the Big-Five personality dimensions: reliability and validity. Eur. J. Personal..

[55] Del Barrio V., Aluja A., García L.F. (2004). Relationship between empathy and the Big Five personality traits in a sample of Spanish adolescents. Soc. Behav. Personal. Int. J..

[56] Judge T., Cable D., Boudreau J., Bretz R. (1995). An empirical investigation of the predictors of executive career success. Pers. Psychol..

[57] Brayfield A.H., Rothe H.F. (1951). An index of job satisfaction. J. Appl. Psychol..

[58] Judge T.A., Locke E.A., Durham C.C., Kluger A.N., Brayfield A.H., Rothe H.F. (1998). Dispositional effects on job and life satisfaction: The role of core evaluations. J. Appl. Psychol..

[59] Sultana R., Yousaf A., Khan I., Saeed A. (2016). Probing the interactive effects of career commitment and emotional intelligence on perceived objective/subjective career success. Pers. Rev..

[60] Yuan L., Tan X., Huang C., Zou F. (2014). mediating effect of job satisfaction on the relationship between emotional intelligence and perceived general health. Soc. Behav. Personal. Int. J..

[61] Miao C., Humphrey R.H., Qian S. (2017). A meta-analysis of emotional intelligence effects on job satisfaction mediated by job resources, and a test of moderators. Personal. Individ. Differ..

[62] Dodangoda H.C., Arachchige B.J.H. (2015). Impact of personality on career success of the employees in the Sri Lankan Banking Sector in Western Province. Hum. Resour. Manag. J..

[63] Seibert S., Kraimer M., Crant M. (2001). What do proactive people do? A longitudinal model linking proactive personality and career success. Pers. Psychol..

[64] Spurk D., Abele A.E. (2011). Who earns more and why? A multiple mediation model from personality to salary. J. Bus. Psychol..

[65] Zhai Q., Willis M., O’Shea B., Zhai Y., Yang Y. (2013). Big Five personality traits, job satisfaction and subjective wellbeing in China. Int. J. Psychol..

[66] Pan J., Guan Y., Wu J., Han L., Fu X. (2017). Internship quality, proactive personality and university graduates job search outcomes. Acad. Manag. Proc..

[67] Li N., Crant M., Liang J. (2010). The role of proactive personality in job satisfaction and organizational citizenship behavior: A relational perspective joint effects of creative self-efficacy, positive and negative affect on creative performance view project. J. Appl. Psychol..

[68] Li M., Wang Z., Gao J., You X. (2017). Proactive personality and job satisfaction: The mediating effects of self-efficacy and work engagement in teachers. Curr. Psychol..

[69] Fuller R., Schoenberger R. (1991). The gender salary gap: Do academic achievement, internship experience, and college major make a difference?. Soc. Sci. Quaterly.

[70] Wang M.-T., Eccles J.S., Kenny S. (2013). Not lack of ability but more choice. Psychol. Sci..

[71] Buser T., Niederle M., Oosterbeek H. (2014). Gender, competitiveness, and career choices. Quaterly J. Econ..

[72] Heckert T.M., Droste H.E., Adams P.J., Griffin C.M., Roberts L.L., Mueller M.A., Wallis H.A. (2002). Gender differences in anticipated salary: Role of salary estimates for others, job characteristics, career paths, and job inputs. Sex Roles.

[73] Willett L.L., Halvorsen A.J., McDonald F.S., Chaudhry S.I., Arora V.M. (2015). Gender differences in salary of internal medicine residency directors: A national survey. Am. J. Med..

[74] Ng T.W.H., Eby L.T., Sorensen K.L., Feldman D.C. (2005). Predictors of objective and subjective career success: A meta-analysis. Pers. Psychol..

[75] Goldberg C.B., Finkelstein L., Perry E.L., Konrad A.M. (2004). Job and industry fit: The effects of age and gender matches on career progress outcomes. J. Organ. Behav..

[76] White A.T., Spector P.E., Martin-Raugh M.P., Kell H.J., Motowidlo S.J. (1987). An investigation of age-related factors in the age-job-satisfaction relationship. Psychol. Aging.

[77] Dohmen T.J. (2004). Performance, seniority and wages: Formal salary systems and individual earnings profiles. Labour Economics.

[78] Chiva R., Alegre J. (2008). Emotional intelligence and job satisfaction: The role of organizational learning capability. Pers. Rev..

[79] Martin-Raugh M.P., Kell H.J., Motowidlo S.J. (2016). Prosocial knowledge mediates effects of agreeableness and emotional intelligence on prosocial behavior. Personal. Individ. Differ..

[80] Jorfi H., Yacco H.F.B., Shah I.M. (2012). Role of gender in emotional intelligence: Relationship among emotional intelligence, communication effectiveness and job satisfaction. Int. J. Manag..

[81] Lee Y.H., Chelladurai P. (2018). Emotional intelligence, emotional labor, coach burnout, job satisfaction, and turnover intention in sport leadership. Eur. Sport Manag. Q..

[82] Judge T.A., Heller D., Mount M.K. (2002). Five-Factor model of personality and job satisfaction: A meta-analysis. J. Appl. Psychol..

[83] Horng J.S., Tsai C.Y., Yang T.C., Liu C.H., Hu D.C. (2016). Exploring the relationship between proactive personality, work environment and employee creativity among tourism and hospitality employees. Int. J. Hosp. Manag..

[84] Mayer J., Salovey P., Caruso D. (2002). Mayer-Salovey-Caruso Emotional Intelligence Test (MSCEIT) Users Manual.

[85] Dacre Pool L., Qualter P. (2012). Improving emotional intelligence and emotional self-efficacy through a teaching intervention for university students. Learn. Individ. Differ..

[86] Sarrionandia A., Garaigordobil M. (2017). Efectos de un programa de inteligencia emocional en factores socioemocionales y síntomas psicosomáticos. Revista Latinoamericana de Psicología.

[87] Meyer G.J., Finn S.E., Eyde L.D., Kay G.G., Moreland K.L., Dies R.R., Eisman E.J., Kubiszyn T.W., Reed G.M. (2001). Psychological testing and psychological assessment: A review of evidence and issues. Am. Psychol..

[88] Lopes P.N. (2016). Emotional intelligence in organizations: Bridging research and practice. Emot. Rev..

